# Number of subjects required in common study designs for functional GABA magnetic resonance spectroscopy in the human brain at 3 Tesla

**DOI:** 10.1111/ejn.14618

**Published:** 2019-12-11

**Authors:** Faezeh Sanaei Nezhad, Caroline A. Lea‐Carnall, Adriana Anton, JeYoung Jung, Emilia Michou, Stephen R. Williams, Laura M. Parkes

**Affiliations:** ^1^ Division of Informatics, Imaging and Data Science University of Manchester Manchester UK; ^2^ Division of Neuroscience and Experimental Psychology University of Manchester Manchester UK; ^3^ School of Psychology University of Nottingham Nottingham UK; ^4^ School of Rehabilitation Sciences University of Patras Patras Greece

**Keywords:** fMRS, GABA, MEGA‐PRESS, power calculation, precision, quantification

## Abstract

Magnetic resonance spectroscopy (MRS) is a research tool for measuring the concentration of metabolites such as γ‐aminobutyric acid (GABA) and glutamate in the brain. MEGA‐PRESS has been the preferred pulse sequence for GABA measurements due to low physiological GABA concentrations, hence low signal. To compensate, researchers incorporate long acquisition durations (7–10 min) making functional measurements of this metabolite challenging. Here, the acquisition duration and sample sizes required to detect specific concentration changes in GABA using MEGA‐PRESS at 3 T are presented for both between‐groups and within‐session study designs. 75 spectra were acquired during rest using MEGA‐PRESS from 41 healthy volunteers in 6 different brain regions at 3 T with voxel sizes between 13 and 22 cm^3^. Between‐group and within‐session variance was calculated for different acquisition durations and power calculations were performed to determine the number of subjects required to detect a given percentage change in GABA/NAA signal ratio. Within‐subject variability was assessed by sampling different segments of a single acquisition. Power calculations suggest that detecting a 15% change in GABA using a 2 min acquisition and a 27 cm^3^ voxel size, depending on the region, requires between 8 and 93 subjects using a within‐session design. A between‐group design typically requires more participants to detect the same difference. In brain regions with suboptimal shimming, the subject numbers can be up to 4‐fold more. Collecting data for longer than 4 min in brain regions examined in this study is deemed unnecessary, as variance in the signal did not reduce further for longer durations.

## Graphical abstract

The impact of acquisition time on variability of GABA data. The figure shows the relative SD of regional GABA estimates across subjects. In general, there is little benefit in collecting data beyond 4 min acquisition duration in terms of improved power to detect group differences.

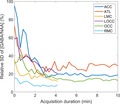

**Abbreviations**
ACC: Anterior cingulate cortex
ATL: Anterior temporal lobe
CRLB: Cramer Rao lower bound
CSF: Cerebrospinal fluid
EEG: Electroencephalogram
fMRI: Functional magnetic resonance imaging
fMRS: Functional magnetic resonance spectroscopy
GABA: γ‐aminobutyric acid
Gln: Glutamine
Glu: Glutamate
GM: Grey matter
HLSVD: Hankel Lanczos Singular Values Decomposition
LMC: Left motor cortex
LOCC: Left occipital cortex
MEGA‐PRESS: Mescher‐Garwood point‐resolved spectroscopy
MRS: Magnetic resonance spectroscopy
NAA: N‐acetylaspartate
OCC: Occipital cortex
RMC: Right motor cortex
ROI: Region of interest
S1: Primary somatosensory cortex
SNR: Signal‐to‐noise ratio
T: Tesla
TE: Echo time
TR: Repetition time
WM: White matter

## 1 INTRODUCTION

Magnetic resonance spectroscopy is a valuable tool used to study brain metabolism in vivo in clinical and healthy human populations. The technique has recently gained popularity in neuroscience research as it allows robust and reliable measurements of glutamate and γ‐aminobutyric acid (GABA), the brain's primary excitatory and inhibitory neurotransmitters, using existing MRI hardware. Research incorporating MRS measurements of GABA has enhanced our understanding of the underlying biochemistry of healthy brain function such as in motor learning (Floyer‐Lea, Wylezinska, Wylezinska, Kincses, & Matthews, 2006; Stagg, 2014), visual stimulation (Lin, Stephenson, Stephenson, Xin, Napolitano, & Morris, 2012; Schaller, Mekle, Mekle, Xin, Kunz, & Gruetter, 2013), pain stimulation (Cleve, Gussew, Gussew, & Reichenbach, 2015; Gussew et al., 2010; Gutzeit et al., 2011) and cognitive function (Lally et al., 2014; Michels et al., 2012). In recent years, there have been several studies measuring changes in the concentration of metabolites as a function of time, either in response to neural activation (Ip et al., 2017; Lin et al., 2012), or immediately pre‐ and post‐intervention (Antonenko et al., 2017; Bachtiar, Near, Near, Johansen‐Berg, & Stagg, 2015), which for the purpose of this paper we term functional magnetic resonance spectroscopy (fMRS). However, progress in this field is hampered by the long acquisition durations required to achieve reasonable spectrum signal‐to‐noise ratio (SNR).

Measuring GABA accurately at 3 T using traditional MRS acquisition protocols is challenging due to its relatively low concentration compared to other metabolites in the brain (Govindaraju, Young, Young, & Maudsley, 2000; Stagg, Bachtiar, Bachtiar, & Johansen‐Berg, 2011). At 3 T, there is spectral overlap of the GABA peaks with much larger peaks such as creatine (Cr) and phosphocreatine (PCr) at 3.02 ppm, glutamate at 2.35 ppm and NAA at 2.02 ppm making quantification of GABA difficult. To overcome this problem, a difference‐editing technique such as MEGA‐PRESS can be used, which collects interleaved spectral acquisitions that differ in their effect on the GABA spin system (Mescher, Merkle, Merkle, Kirsch, Garwood, & Gruetter, 1998). MEGA‐PRESS is the most commonly used method to quantify GABA at 3 T and has been shown to be robust and reliable (Mullins et al., 2014; Gorman, Michels, Michels, Edden, Murdoch, & Martin, 2011; Oz et al., 2006; Shungu et al., 2016; Yasen, Smith, Smith, & Christie, 2017).

The current practice of GABA measurement in the brain using MEGA‐PRESS compensates for the low GABA signal due to low physiological GABA concentrations with longer acquisition times (7–10 min) and large voxel sizes (generally 3 × 3 × 3 cm^3^) (Mullins et al., 2014; Puts & Eden, 2012). This makes functional measurements difficult to interpret as neurophysiological changes generally occur at faster timescales than this. Reducing the time required for robust GABA quantification would also be advantageous for general MRS research as current GABA acquisition durations can be prohibitive in terms of total time required in the scanner, especially when dealing with populations for whom this time must be minimised. Variance in the GABA measurement is the only factor of relevance in determining subject numbers (or acquisition duration or voxel size) required to detect a particular effect size; as is clear from Equation 3. It is therefore important that we understand the relationship between variance in the GABA‐edited signal and acquisition duration.

In this paper, we examine GABA‐edited data collected from 6 different brain regions using MEGA‐PRESS at 3 T to test whether longer acquisition durations are warranted to increase the SNR and so improve precision. We demonstrate that at the group level, variance in the data reaches a point at which it does not reduce any further with acquisition time and so collecting data for longer than this may not be useful. We estimate the sample size and the necessary acquisition duration required to detect statistically significant concentration changes in GABA (estimated as a ratio relative to NAA) for between‐groups and within‐session study designs in the relevant brain regions with an assumed voxel size of 27 cm^3^ (3 × 3 × 3 cm). The within‐session subject numbers will inform the design of fMRS studies and the between‐group analyses will be useful for more general MRS research.

## 2 METHODS

All acquisitions were performed on a 3 T MRI scanner (Philips Achieva, Best, the Netherlands), using a body coil for transmission and a 32‐channel head coil for signal reception.

### 2.1 Subjects and data acquisition

**Figure 1:**
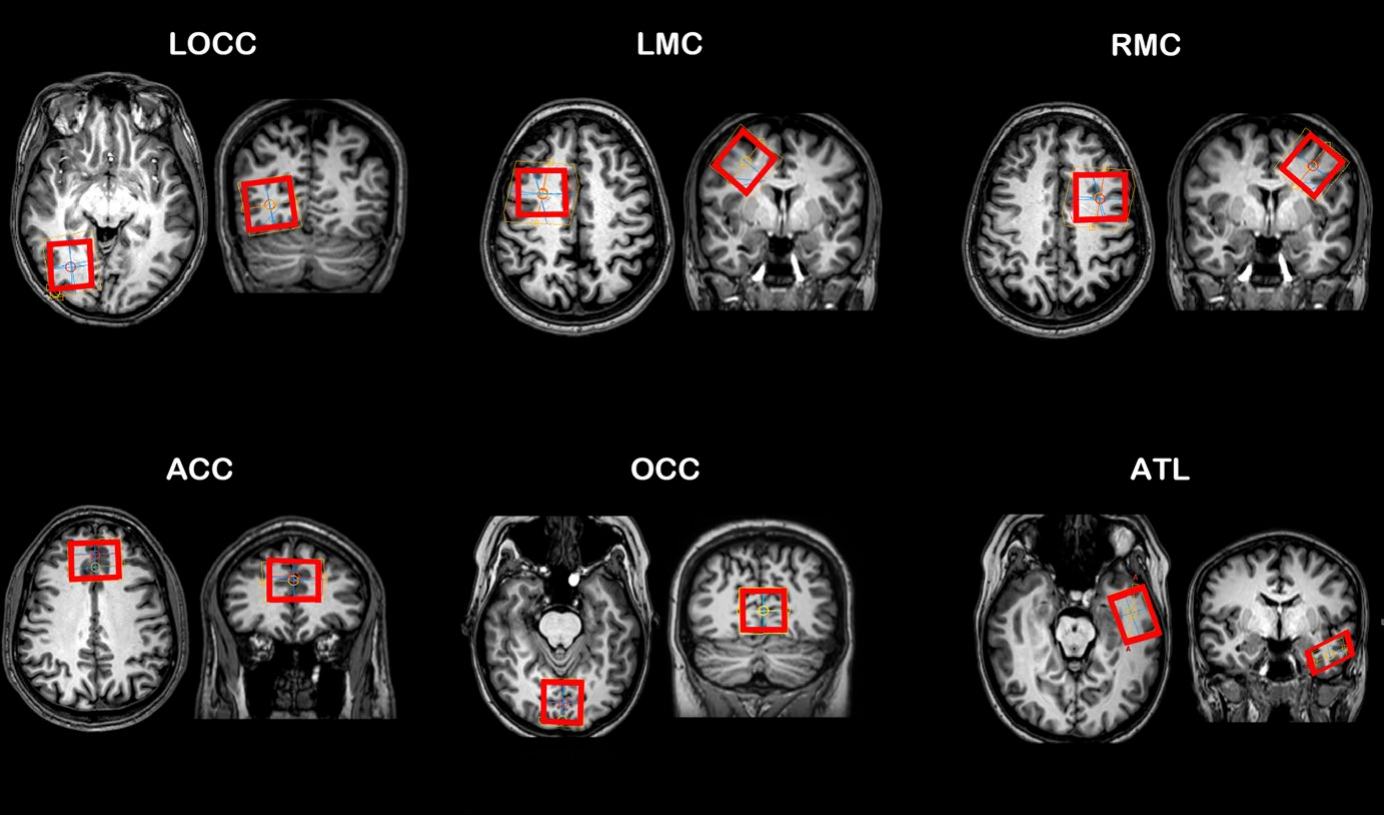
Voxel locations are given for each of the regions analysed in the main study [Colour figure can be viewed at http://wileyonlinelibrary.com]

83 GABA‐edited spectra were acquired during rest using MEGA‐PRESS from 41 healthy volunteers (20 female, 21 male, age range 20–55 years old, mean age 29.4 ± 9.2) who all gave written informed consent in accordance with procedures approved by University of Manchester Ethics Committee (Refs 04/Q1405/66, 12049, 14/NW/0298, 08/H1004/93). For all acquisitions, a 1‐mm isotropic T_1_‐weighted image was acquired to aid the positioning of the ^1^H MRS voxel and to correct for grey matter concentration. Ten of the volunteers had ^1^H MRS measurements in three different brain regions with a voxel size of 32 × 32 × 32 mm^3^ as follows: left occipital cortex (LOCC), left motor cortex (LMC) and right motor cortex (RMC). Nine of the volunteers had measurements in the anterior cingulate cortex (ACC) with a voxel size of 35 × 40 × 20 mm^3^ and 22 had measurements in the occipital cortex (OCC) with a voxel size of 30 × 30 × 30 mm^3^ and the anterior temporal lobe (ATL) with a voxel size of 35 × 25 × 15 mm^3^ (see Figure 1). All participants were scanned at rest and were instructed to remain with eyes open and the lights in the scanning room were switched on.

The GABA‐edited MEGA‐PRESS spectra (TE/TR = 70/2000 ms, 1,024 samples, 2kHz bandwidth) were acquired in consecutive blocks of 4 averages when the MEGA pulse was set at 1.89 ppm (MEGA‐on), referred to as a single dynamic, followed by four averages of MEGA pulse set at 7.6 ppm (MEGA‐off). The dynamics were then repeated in an interleaved manner. Most of the peaks in the spectrum are unaffected by the editing pulses, therefore subtraction of the averaged ON from the OFF spectra results in removal of all these peaks, leaving only those peaks targeted by the editing pulses (Mullins et al., 2014). The final edited spectrum contains all signals close to the 1.89 ppm editing pulse, signal from the coupled GABA signal at 3 ppm, as well as the coupled signal at 3.75 ppm for the combined measures of glutamate and glutamine (Glx) and co‐edited macromolecule signals.

LMC, LOCC and RMC voxels had 32 dynamics in total (acquisition time 4 min 16 s); ACC, ATL, OCC had 74 dynamics in total (acquisition time 9 min 52 s). The water suppression method had an excitation with a window of 140 Hz, and the shimming was second‐order pencil beam (FASTMAP), as described previously (Sanaei Nezhad et al., 2017). The N‐acetylaspartate (NAA) peak at 2.02 ppm was used for frequency referencing.

### 2.2 Metabolite quantification

Analysis of the spectroscopic data to quantify GABA was performed in jMRUI 6.0 software (Stefan et al., 2009) using QUEST (Ratiney et al., 2005), a non‐linear least squares fitting algorithm. QUEST is a time‐domain algorithm which fits a weighted combination of metabolite signals directly to the data. An initial metabolite basis set, used as prior knowledge for QUEST quantification, was obtained by scanning single metabolite phantoms: NAA, Glu, Gln and GABA. For accurate frequency referencing and phase estimation of each in vivo spectrum, pre‐processing was performed as follows. First, the dynamics which had their MEGA frequency set at 7.6 ppm (MEGA‐off) were summed. The NAA peak was set as 2.02 ppm and its phase was estimated using AMARES (Vanhamme, van den Boogaart, van den Boogaart, & Van Huffel, 1997), a resonance‐by‐resonance quantification method based on a non‐linear least squares algorithm. Since the NAA peak in GABA‐edited MEGA‐PRESS has a 180° phase shift relative to the GABA peak at 3 ppm, the MEGA‐PRESS phase was fixed at the NAA phase minus 180°. Any residual water peak was removed using Hankel Lanczos Singular Values Decomposition routine in jMRUI. Finally, all the dynamics were summed to give the GABA‐edited spectrum. The implementation of MEGA‐PRESS on the scanner is such that there is a phase shift of 180° between the MEGA‐on and MEGA‐off dynamics so that the GABA signal is always positive.

#### 2.2.1 Concentration calculation

GABA concentration was estimated using the NAA signal as a concentration reference, according to the following equation:

(1) $$\left[\text{GABA}\right]=\frac{S_{\text{GABA}}}{2*S_{\text{NAA}}}\times \frac{n_{\text{NAA}}}{n_{\text{GABA}}}\times \left[\text{NAA}\right]\times \frac{1}{E}$$

where *S*_GABA_ and *S*_NAA_ are the raw GABA and NAA signals, respectively. The factor 2 is to correct for the suppression of the NAA peak in the MEGA‐on spectra. The *n*_GABA_ and *n*_NAA_ are the number of protons in the GABA and NAA molecules. [NAA] is the concentration of NAA in human brain, which was assumed to be 8 mM in all calculations. *E* is the editing efficiency, defined as the ratio of the edited GABA signal intensity compared to the non‐edited intensity in a sample which only contains GABA. This was set to 0.5 (Mullins et al., 2014) though this does not take account of signal loss due to chemical shift displacement effects when spectroscopic editing is performed with slice‐selective pulses (Slotboom, Mehlkopf, Mehlkopf, & Bovee, 1994). This effect is sequence‐dependent and constant across all the studies reported here so has not been factored in.

#### 2.2.2 Quality control

All spectra were subjected to quality control assessment as per (Sanaei Nezhad et al., 2017). Briefly, GABA SNR, NAA linewidth and Glx Cramer–Rao lower bound (CRLB) were calculated for every data set with an acquisition duration of 4 min 16 s of data and the spectra passed quality control if the following conditions were met:

1. NAA linewidth and Glx CRLB are both less than the mean plus 2 standard deviations of all the data within that region.
2. GABA SNR is greater than the mean minus 2 standard deviations of all data within that region.

Note that each spectrum is judged on the basis of the average data quality within the region it was acquired, rather than using the cut‐offs suggested in Sanaei Nezhad et al. (2017). This was implemented to provide sample size information for both high‐ and low‐quality data. On this basis, 8 spectra were excluded from further analysis leaving 75 spectra in total. Please see Table 1 for a breakdown by region.

**Table 1:** GABA data across subjects are reported in different regions of interest. Mean across subjects of GABA concentration, the grey and white matter percentage within the voxel, and the spectral quality factors are given as mean ± *SD* at 4 min 16 s acquisition time

Region (rejected spectra)	Voxel Size cm^3^	[GABA/NAA]	GM%	WM%	GABA SNR	NAA LW (Hz)	Glx CRLB(%)
ACC *N* = 9 (2)	28	4.2 ± 0.8	43.4 ± 2.6	46.1 ± 2.5	3.1 ± 0.8	5.6 ± 0.5	8.5 ± 1.3
ATL *N* = 22 (2)	13.1	2.1 ± 1.0	50.9 ± 4.2	46.8 ± 4.7	1.2 ± 0.4	12.1 ± 4.0	36.6 ± 24.7
LMC *N* = 10 (0)	32.8	3.0 ± 0.9	31.0 ± 2.7	62.2 ± 4.3	2.6 ± 0.6	6.3 ± 1.0	18.0 ± 22.0
LOCC *N* = 10 (0)	32.8	3.3 ± 0.6	42.7 ± 3.0	52.8 ± 6.0	2.5 ± 0.4	6.8 ± 1.5	24.0 ± 46.0
OCC *N* = 22 (4)	27	3.8 ± 0.5	54.2 ± 6.0	32.2 ± 10.1	3.7 ± 0.9	5.9 ± 0.6	7.3 ± 2.0
RMC *N* = 10 (0)	32.8	3.4 ± 0.7	31.7 ± 4.2	61.0 ± 6.0	1.9 ± 0.9	5.9 ± 0.3	12.0 ± 5.0

### 2.3 Statistical analysis

#### 2.3.1 Calculation of variance

The variance across subjects determines the power in a between‐groups study design. As variance depends on acquisition time, we calculated the mean and variance in GABA concentration across subjects as a function of acquisition duration for each region of interest (ROI). To do this, spectra were averaged for each participant up to the specified acquisition duration and GABA concentration was quantified as a ratio to NAA. In order to remove the impact of any physiological change in GABA concentration over the duration of the acquisition, individual spectra were sampled (without replacement) from the full set such that the number of averages equated to the acquisition duration of interest. These spectra were then used to determine the mean and *SD* for each duration and ROI.

The variance between repeat measurements in the same person determines the power in a within‐session study design. The within‐session variance was estimated by splitting the data either into two segments (of 2 min 8 s or 4 min 16 s duration) or, if enough data were acquired, into four segments (of 2 min 8 s duration) to yield two or four measurements within a single scan session for each person. For data sets that had only two measurements per subject, the Bland–Altman variance estimation was used as below (Bland & Altman, 1996):

(2) $$\sigma^{2}=\frac{1}{2n}\sum_{i=1}^{n}d_{i}^{2}$$

where *n* is the number of subjects and *d_i_* is the difference between the two measurements for subject *i*. For datasets where there were four measurements, the per‐person variance was averaged across participants. As for the between‐groups analysis, individual spectra were sampled at random from the complete set in order to make up the data segments of different lengths for each participant.

For ease of comparison between regions, we calculated the relative standard deviation (*SD*), defined as the *SD* normalised by the mean (here we used the mean as calculated at 4 min 16 s as variance in the concentration did not decrease past this point). We further normalised the relative *SD* by multiplying by the size of the voxel (in cm^3^) and dividing by 27 to give a measurement of relative *SD* for a voxel of size of 27 cm^3^. This was to be able to compare our results per unit voxel size. This assumes that the *SD* of the signal is inversely proportional to the voxel size (Macovski, 1996) and is also consistent with the expectation that the noise in a voxel is independent of voxel size (as it is due to resistive coupling of the whole receiver coil with the sample), whereas signal is directly proportional to the voxel volume.

#### 2.3.2 Power calculation to determine sample size

Power calculations were performed in each ROI to determine the number of subjects (*N*) required to detect a given effect size (eff). The following equation was used (Noordzij et al., 2010; Zar, 1996):

(3) $$N=\frac{2\sigma^{2}{\left(Z_{1-\alpha /2}+Z_{1-\beta }\right)}^{2}}{{\text{eff}}^{2}}$$

where $Z_{1-\alpha /2}$ is the Z score (number of standard deviations from the mean) related to the significance level which was considered as 0.05 ($Z_{1-\alpha /2}=1.96$). $Z_{1-\beta }$ is the Z score of the detection power which was considered as 80% ($Z_{1-\beta }=0.8416$). The variance $\sigma^{2}$ was estimated as described in the section above (we used the square of the relative standard devation for a standard voxel size of 27 cm^3^) and the power calculations performed for both between‐group and within‐session study designs for a range of effect sizes.

### 2.4 Regional comparisons

In order to test whether there were regional differences in the GABA/NAA concentration, a 1‐way analysis of variance test (ANOVA) was performed on mean concentrations at 4 min 16 s across all participants using R (R Core Team, 2017). Similar analysis were performed to test for regional differences in the percentage of grey and white matter.

## 3 RESULTS

### 3.1 Calculation of variance

**Figure 2:**
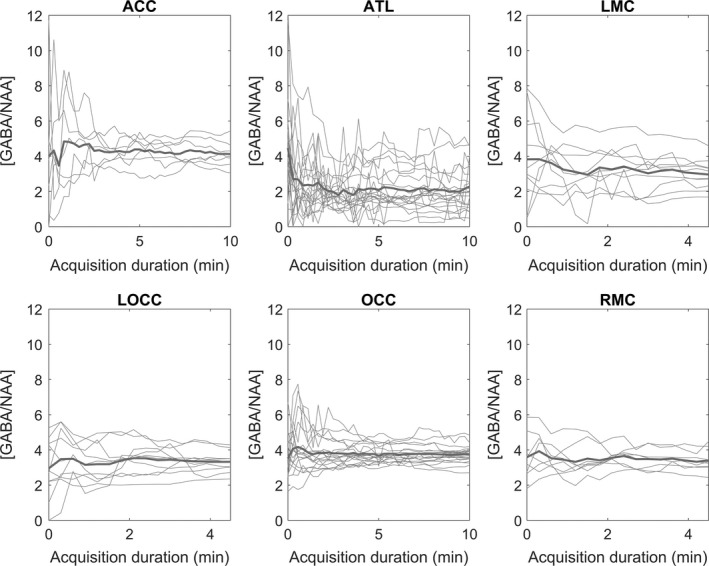
GABA/NAA concentration measurement is shown for each participant as the acquisition duration increases for each ROI with mean in bold

**Figure 3:**
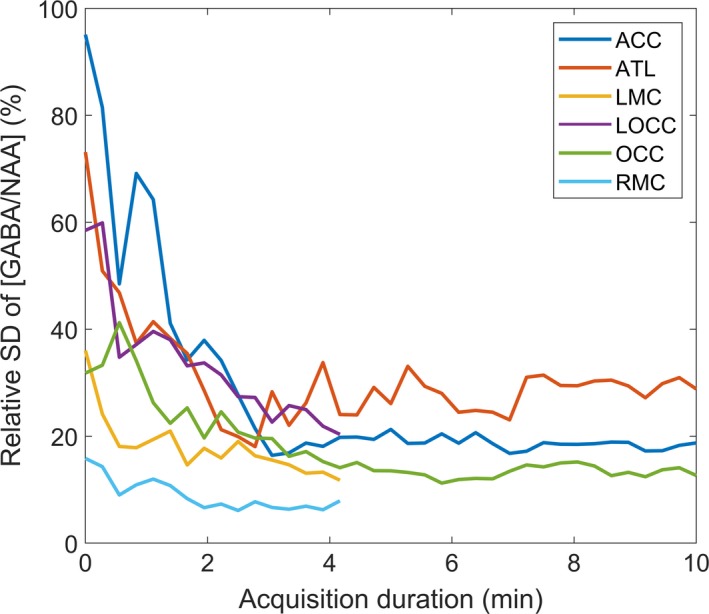
The relative *SD* is given for a standard voxel size of 27 cm3 per ROI as a function of acquisition duration. It can be seen that the variance does not reduce any further for acquisition times greater than 4 min [Colour figure can be viewed at http://wileyonlinelibrary.com]

GABA concentration measurements with increasing acquisition duration are shown for each brain region for each participant in Figure 2. Figure 3 shows the relative *SD* of the GABA concentration measurements with increasing acquisition duration for each brain region for a standard voxel size of 27 cm^3^. The mean concentration of GABA across subjects at 4 min 16 s is within 13% of the final value for all regions with longer acquisitions, suggesting reasonable accuracy at this duration. The variance does not continue to decrease after 4 min 16 s, suggesting that this is sufficient time for precise GABA concentration measurements in all ROI’s shown here with no benefit of collecting data for longer.

**Figure 4:**
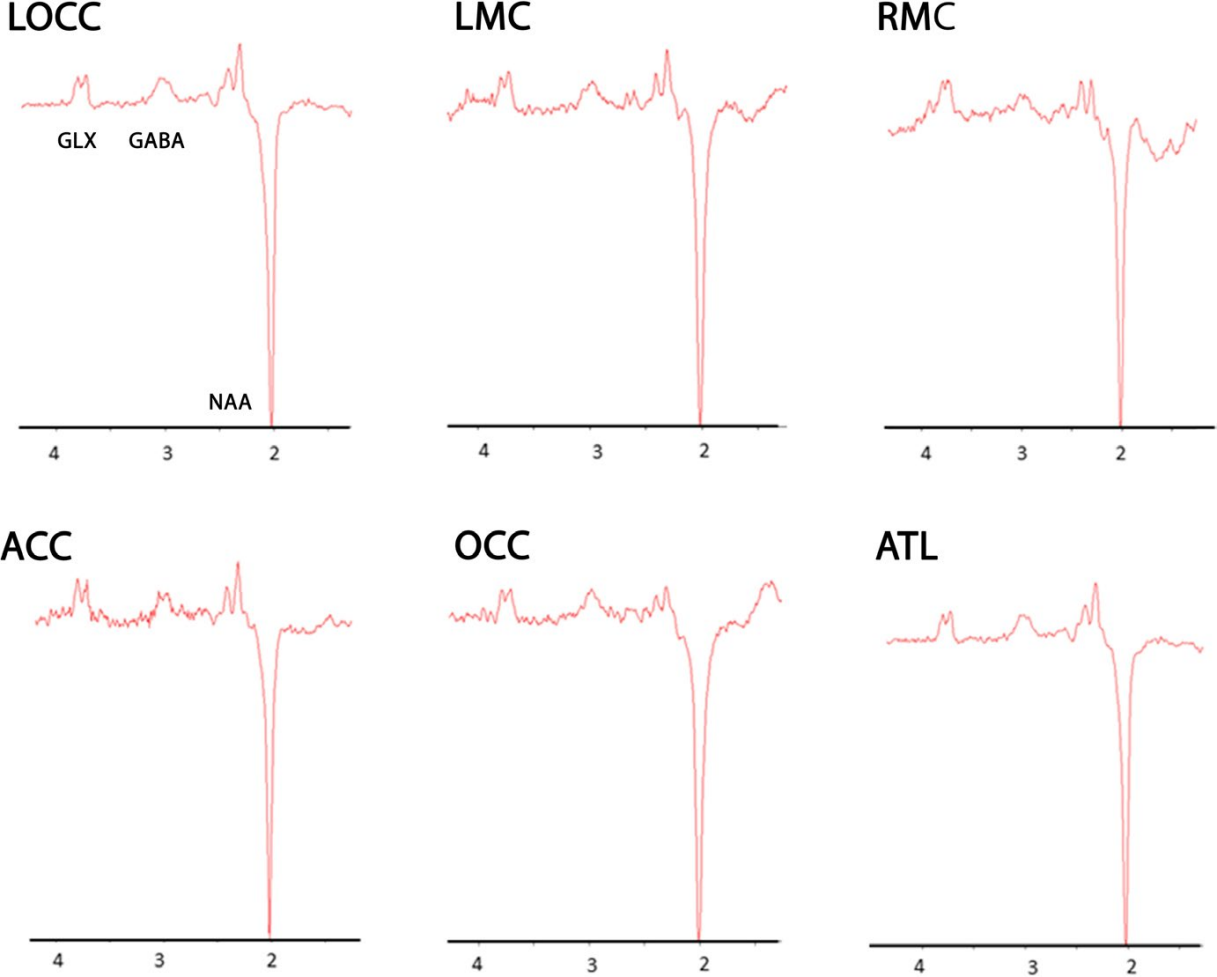
A sample spectrum with 4 min 16 s acquisition time in each of the regions analysed. GABA, Glx and NAA are labelled on the first spectrum [Colour figure can be viewed at http://wileyonlinelibrary.com]

The mean and relative *SD* of GABA/NAA concentration for a standard voxel size of 27 cm^3^, the grey and white matter percentage within the voxel, and the spectral quality factors are given for each region in Table 1. Since the variance of the GABA concentration does not continue to decrease after 4 min 16 s, values are reported at this duration for all voxels. Figure 4 illustrates a sample spectrum in each region.

### 3.2 Sample sizes

**Figure 5:**
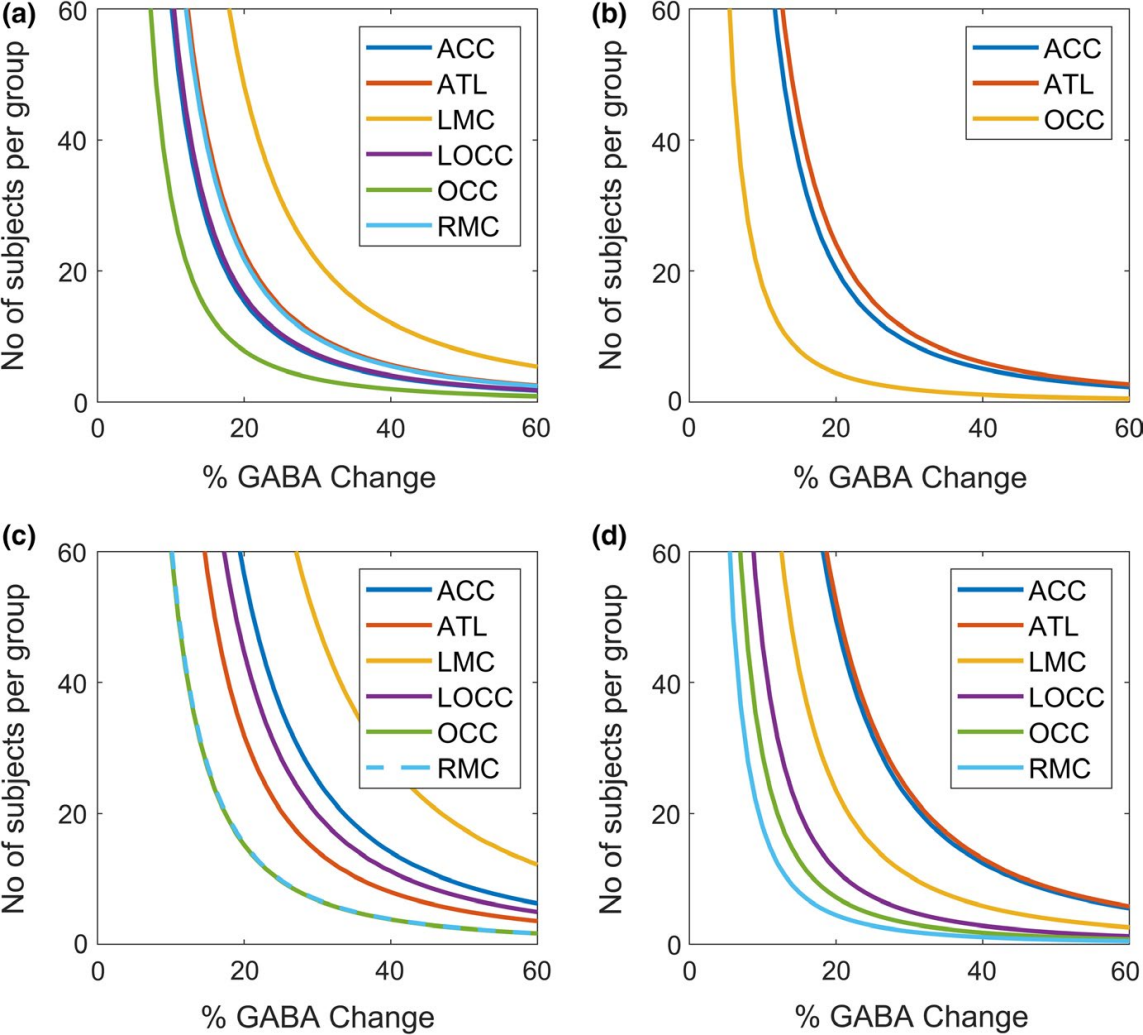
The sample size required to detect a given effect size in each region of interest is plotted for (a) between‐group design for duration of 4 min 16 s, (b) within‐session design for duration of 4 min 16, (c) between‐group design for duration of 2 min 8 s (d) within‐session design for duration of 2 min 8 s. Equation 3 was used to calculate the required number of subjects [Colour figure can be viewed at http://wileyonlinelibrary.com]

Sample sizes for between‐group and within‐session study design were calculated using Equation 3. Figure 5 demonstrates that the number of subjects required to detect a given effect size varies according to the region of interest. The number of subjects required to detect a 15% change of GABA concentration in each region of interest is reported in Table 2 for each region, study design and duration investigated (assuming a standard voxel size of 27 cm^3^). Here, we use 15% as an illustration based on a number of previous studies who have found a change in GABA in this range in response to intervention, for example (Cleve et al., 2015; Padulo et al., 2016). As expected it can be observed that for the same effect size, a larger number of subjects is required for between‐group studies compared to a within‐session study, and the longer the acquisition time, the fewer subjects that are needed.

**Table 2:** The number of subjects required to detect a 15% change of GABA in each region of interest using a standard 27 cm^3^ voxel is reported for each region, study design and duration investigated

Region	Between‐group 4 min 16 s	Within‐session 4 min 16 s	Between‐group 2 min 8 s	Within‐session 2 min 8 s
ACC	27	36	100	88
ATL	40	43	57	93
LMC	86	NA	195	42
LOCC	29	NA	79	20
OCC	14	8	27	13
RMC	39	NA	27	8

### 3.3 Regional comparisons

We performed a 1‐way ANOVA on mean GABA/NAA concentrations at 4 min 16 s across all ROIs and found a significant effect of ROI on GABA/NAA concentrations (*F* = 7.52, *p* = .008), we note that this effect may be due to variations in either metabolite.

We performed a 1‐way ANOVA on GM and WM percentages across all ROIs and found no effect of ROI on GM percentages within the voxels (*F* = 0.23, *p* = .64) or WM (*F* = 1.73, *p* = .19).

## 4 DISCUSSION AND CONCLUSIONS

The variance measurements indicate that the implementation of an fMRS study using GABA‐edited MEGA‐PRESS is feasible in a relatively small group size (<40), provided that a minimum acquisition time of 4 min 16 s is allocated per measure in the regions we considered except LMC. For shorter acquisition times the number of subjects required increases depending on the region, but the ability to detect functional changes in a within‐session design over a 2‐min duration is still feasible. For acquisition times below approximately 2 min, we found that group‐level variance was high (Figure 3), reducing the possibility of detecting group‐wise differences in GABA concentration that occur over this timescale.

The decrease in relative *SD* with increasing acquisition duration (Figure 3) initially follows approximately the relation predicted by theory that *SD* is inversely proportional to the square root of acquisition duration (Macovski, 1996), that is to halve the *SD*, acquisition duration must be increased four‐fold. However, this holds only up to a certain point with no clear gain beyond 4 min of acquisition. Beyond this point, it seems that true biological variability across subjects limits further gain. Therefore, to have power to detect smaller effect sizes, either subject numbers or voxel volume must be increased. It should be noted that the minimum detectable effect size depends on the inverse root of the number of subjects (Equation 3) but also on the inverse of voxel size, so to halve the effect size either the voxel size would need to be doubled, or the number of subjects would need to be quadrupled. The limited acquisition duration of our data did not allow us to test beyond 4 min 16 s for the within‐session design so it is unclear whether additional gains can be made with longer acquisition duration in this case.

The ATL region shows the highest variance at 4 min 16 s, even after correcting for the smaller voxel size of this region. In ATL, the SNR value is the lowest among all studied regions which in part reflects a lower GABA concentration (see Table 1), though the lower SNR is more than expected simply from a lower concentration. Additionally, the other quality control factors NAA linewidth and Glx CRLB, were highest in the ATL (see Table 1). Hence, the variance may be attributed mainly to lower spectral quality rather than greater biological variability. It is worth stating that this region is particularly hard to shim due to its proximity to the sinus. Note that only data from the ACC and OCC pass the strict spectral quality test as set out in (Sanaei Nezhad et al., 2017), that is SNR > 1.0, NAA linewidth < 8 Hz and Glx CRLB < 16%. These limits are suggested for quantification of Glutamate and Glutamine and so may be too strict for accurate assessment of GABA but nevertheless serve as a guide for very high‐quality data. The number of subjects required to detect a 15% changes in GABA concentration in ACC is however markedly greater than for OCC, suggesting greater biological variability across people and time for ACC in comparison to OCC. However, the relatively low number of subjects with data for the ACC region may make this interpretation unreliable.

The significant GABA/NAA signal difference between the 6 ROIs suggests a regional neurotransmitter concentration variation. This has also been found in different studies mapping GABA levels in the brain (Gaetz et al., 2014; Harada, Kubo, Kubo, Nose, Nishitani, & Matsuda, 2011; Zhu, Edden, Edden, Ouwerkerk, & Barker, 2011). The GM and WM composition can also play a role in this variation; however, the similar GM content and yet different GABA concentration in ROIs such as ACC and LOCC or OCC and ATL weaken this hypothesis.

The power calculations show that at 3 T, detecting a GABA difference lower than 5% in all investigated regions, would not be possible with a reasonable number of subjects (<50) (see Figure 5). This is an important finding for studies that hypothesise a low concentration change in GABA. However, for an effect size of around 15%, we predict much more reasonable sample sizes (between 8 and 43 depending on the region, excluding the LMC) which is evidenced by recent literature (Cleve et al., 2015; Padulo et al., 2016). The power calculations in this paper are limited to only six regions in the brain; however, it is logical to assume a similar behaviour in adjacent regions with similar spectra quality.

In this study, the within‐subject variability was assessed by randomly sampling individual spectra from the entire acquisition, as described in the Methods. More typically, within‐subject variability is measured by sampling from the same subject during different scan sessions separated by a day or more. Introducing a time interval and repositioning between repeat scanning sessions will likely increase the variability of repeat measurements and hence increase the suggested sample sizes. We chose our within‐session approach to provide appropriate numbers for a true “*functional”* MRS experiment in which there is a manipulation altering GABA within the same scanning session. The subject numbers for the within‐subject design are not greatly different than those for the between‐subject design, suggesting that the variability in GABA estimates within the same person from session to session is not markedly smaller than the variation from person to person.

The results of this study are only based on the basic averaging method for signal acquisition, however statistical resampling techniques such as bootstrapping or jackknife could provide more information about the natural variability of the signal. It is also important to note that GABA concentration will reflect the ongoing neural activity and hence it is important to give consistent instructions to participants during scanning in order to reduce this potential variability. In this study, we simply instructed the participants to remain still with eyes open but, depending on the brain region, it may be worth considering additional constraints such as focussing on a fixation cross or providing a low‐level task.

This study illustrates that fMRS measurement of GABA with MEGA‐PRESS pulse sequence at 3 T is feasible. Practical graphs and power calculations in 6 different brain regions are presented and can be used in research designs. We demonstrate that the acquisition time and the region of interest plays an important role on the minimum effect size that can be detected with a fixed number of subjects.

## CONFLICT OF INTERESTS

The authors have no competing interests to declare.

## AUTHOR CONTRIBUTIONS

FSN, SW, LP designed the study. FSN, CLC, JJ, EM, AA, SW, LP collected the data. FSN, SW, LP analysed the data. FSN, CLC, LP, SW wrote the paper.

## Data Availability

The data can be found at 10.6084/m9.figshare.9878843.
